# Cancer-Related Non-Bacterial Thrombotic Endocarditis Presenting as Acute Ischemic Stroke

**DOI:** 10.7759/cureus.14953

**Published:** 2021-05-11

**Authors:** Pramod Savarapu, Basel Abdelazeem, Sakiru Isa, Nischit Baral, Mustafa Hassan

**Affiliations:** 1 Internal Medicine, McLaren Flint/Michigan State University, Flint, USA; 2 Cardiology, Michigan State University College of Human Medicine, Flint, USA

**Keywords:** stroke, pancreatic-biliary cancer, non-bacterial thrombotic endocarditis, marantic endocarditis

## Abstract

Non-bacterial thrombotic endocarditis (NBTE), also known as marantic endocarditis, is a rare form of noninfectious endocarditis presenting with thromboembolism including ischemic cerebral stroke. It is mostly associated with advanced malignancy and characterized by the presence of sterile vegetation on heart valves. The diagnosis is usually based on vegetations seen on an echocardiogram, with negative blood cultures suggesting a noninfectious etiology. The treatment for this condition includes systemic anticoagulation. In this report, we discuss the case of a 61-year-old Caucasian female who presented to our facility with an ischemic stroke. She was found to have vegetations on the mitral valve with negative blood cultures. Further studies revealed metastatic pancreatic cancer. The patient's condition improved following in-hospital management, and she was discharged home for outpatient follow-up and treatment. Through this report, we highlight the importance of considering a search for malignancy in patients presenting with these clinical features. Early diagnosis and prompt management are critical to reduce the complications of NBTE and improve the patients' quality of life.

## Introduction

Non-bacterial thrombotic endocarditis (NBTE) or marantic endocarditis is a rare form of noninfectious endocarditis presenting as thromboembolism; it mainly causes ischemic stroke, myocardial infarction, pulmonary thromboembolism, acute intestinal ischemia, and splenic infarction [1]. It is characterized by the presence of sterile vegetation, predominantly on the mitral and aortic valves due to platelet and fibrin deposits [2]. This clinical syndrome is associated with advanced malignancy such as pancreatic, lung, and ovarian cancers or autoimmune diseases such as systemic lupus erythematosus [3]. Patients with pancreatic cancer are at a higher risk of stroke, which requires efficient follow-up, especially during the first six months after the cancer diagnosis [4]. A cohort study conducted in Taiwan undertook a comparison between patients with pancreatic cancer but no history of stroke and patients without cancer and stroke from 2000 to 2009, and followed them up for three years, and concluded that a patient with pancreatic cancer had two to three-fold higher incidence of stroke than controls [4]. The prevalence rate of marantic endocarditis as an etiology for stroke in this population is unknown. In this report, we describe a case of marantic endocarditis presenting as acute ischemic stroke and venous thromboembolism. The workup of the patient revealed a new diagnosis of metastatic pancreatic adenocarcinoma.

## Case presentation

The patient was a 61-year-old Caucasian female with a medical history of insulin-dependent diabetes mellitus, essential hypertension, dyslipidemia, and hypothyroidism, who had been referred from another hospital with slurred speech and right-sided limb weakness for about eight hours. She had no history of stroke. Her blood pressure on admission was 160/90mmHg with a heart rate of 95 beats per minute; she was not febrile. On examination, she was found to be aphasic with right-sided hemiparesis.

Initial laboratory studies showed mild anemia with a hemoglobin of 10 g/dL. Electrocardiography (EKG) showed normal sinus rhythm. At first, a non-contrast CT scan was negative for any infarctions, but MRI showed multiple small areas of infarct scattered throughout the supratentorial as well as the infratentorial brain. CT angiography (CTA) showed occlusion of the distal M1 segment of the left middle cerebral artery (MCA). After the administration of tissue plasminogen activator (tPA), she was transferred to our hospital for mechanical thrombectomy as she had persistent neurological deficits.

**Figure 1:**
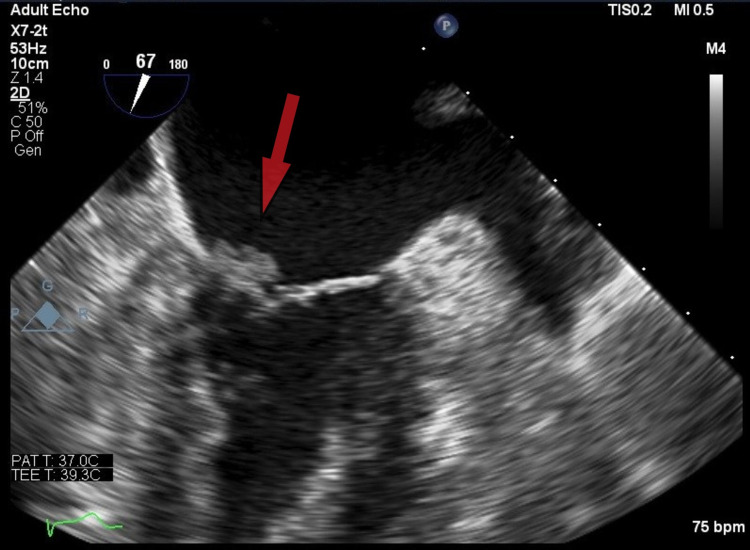
Transthoracic echocardiogram showing vegetations on the anterior mitral valve leaflet (arrow)

The patient underwent endovascular reperfusion and thrombectomy and was subsequently transferred to the ICU. Venous ultrasound showed bilateral lower extremity deep vein thrombosis (DVT). A contrast-enhanced CT scan of the chest was positive for right subsegmental pulmonary embolism. Anticoagulation was withheld in view of the risk of hemorrhagic conversion post thrombectomy. Vascular surgery placed an inferior vena cava (IVC) filter. Transthoracic echocardiogram (TTE) (Figure 1) and transesophageal echocardiogram (TEE) showed a mobile density of the anterior mitral valve leaflet suspicious for vegetations. Bubble study with agitated saline on echo was negative. Blood cultures were also negative. Upon further investigation, carbohydrate antigen 19-9 (CA19-9) was found to be significantly elevated. A contrast-enhanced CT scan of the abdomen revealed pancreatic cancer with liver metastasis. In light of negative blood cultures, mitral valve vegetations on echocardiogram, and acute ischemic stroke, a diagnosis of marantic endocarditis was made.

During the course of the hospital stay, the patient’s condition, including her speech, improved. After discussion with the family about the goals of her care, a palliative approach was determined to be pursued. The patient was discharged for outpatient follow-up with oncology.

## Discussion

NBTE, also known as marantic endocarditis, was first described by Zeigler in 1888. NBTE is a rare condition characterized by the presence of sterile vegetations on the cardiac valves, mainly mitral and aortic valves. These vegetations comprise thrombi intermixed with strands of fibrin, platelets, mononuclear cells, and immune complexes [5]. The vegetations occlude the arterial tree when dislodged. The central nervous system is a frequent site of embolization, leading to ischemic stroke in patients [6,7]. About half of patients with NBTE present with thromboembolic phenomena including stroke [8]. Stroke as an initial presentation of NBTE has been reported in patients with advanced-stage pancreatic adenocarcinoma and non-small cell lung cancer [9,10].

In patients with malignancy, the interaction between macrophages, monocytes, and malignant cells releases interleukins, tumor necrosis factor, and tissue factor causing endothelial damage, which provides a surface for initiation of platelet aggregation and subsequent hypercoagulability and thrombogenesis [2,6,11]. Diagnosis can be made using TTE, or TEE if the diagnosis is missed on TTE. TEE has proven to be the superior technique and must be obtained to confirm the diagnosis [12].

The treatment mainly consists of treating the underlying malignancy or associated condition in addition to initiating systemic anticoagulation. Low molecular weight heparin should be used for anticoagulation and the patient should be placed on lifelong anticoagulation as recurrent thromboembolism has been reported in some patients after its stoppage [13].

Surgery has a limited role in the management, and there are no specific guidelines related to surgery for patients with NBTE. However, surgery can be considered in patients with thromboembolic events despite undergoing anticoagulant therapy, acute heart failure, or acute valvular rupture. The risk and benefits of surgery should be clearly explained to the patient and the family in the context of the underlying disease prognosis and life expectancy [1,6,14].

In the present case, the patient presented with ischemic stroke, the most common initial clinical presentation of NBTE as discussed above. The patient also had DVT and acute pulmonary embolism. This presentation brings to the fore the susceptibility of patients with cancer to venous and arterial thromboembolic phenomena. These phenomena often result from a cascade of endothelial damage and platelet activation. As demonstrated above, it could represent the initial presentation of some malignancies.

## Conclusions

We presented the case of a patient with marantic endocarditis who initially received medical attention due to an acute ischemic stroke. Further evaluation revealed metastatic pancreatic cancer. This case emphasizes the importance of considering underlying malignancy in a patient with an initial presentation of NBTE. Early diagnosis and treatment are critical to reduce the complications of NBTE and improve the patients' quality of life.
